# Abdominal wound dehiscence after appendectomy during pregnancy treated by negative pressure wound therapy with subsequent vaginal delivery: A case report and literature review

**DOI:** 10.1002/ijgo.16155

**Published:** 2025-01-18

**Authors:** Jan Zapletal, Borek Sehnal, Radim Dvorak, Miroslav Drienko, Radovan Vlk, Michael J. Halaska, Lukas Rob

**Affiliations:** ^1^ Department of Obstetrics and Gynecology, Third Faculty of Medicine Charles University and University Hospital Kralovske Vinohrady Prague 10 Czech Republic; ^2^ Department of Surgery, Third Faculty of Medicine Charles University and University Hospital Kralovske Vinohrady Prague 10 Czech Republic

**Keywords:** appendicitis, negative pressure wound therapy, obstetrics, pregnancy, surgical site infection, vacuum‐assisted closure system, wound dehiscence

## Abstract

Negative pressure wound therapy (NPWT) is a very effective method in the treatment of dehiscent, infected, and non‐healing wounds. Difficult wound healing occurs especially in late pregnancy due to the rapid enlargement of the uterus and the constantly increasing tension of the entire abdominal wall. In cases of dehiscence of the surgical wound during pregnancy, proper subsequent treatment is needed, where it is necessary to consider the safety of the mother as well as the fetus. We report the case of a 30‐week pregnant patient who was surgically treated for acute appendicitis in pregnancy with an open appendectomy approach. Postoperative complications resulted in wound dehiscence with complete defect in fascia, which was treated with negative V.A.C. ATS® Therapy System. The therapy was started in the 30th week of pregnancy and continued until delivery with regular check‐ups and regular redressing of the vacuum‐assisted closure (VAC) system. At 38 weeks of pregnancy, the patient delivered vaginally with continued VAC therapy in situ. The final suture took place 3 days after vaginal delivery. Non‐healing wounds with abdominal wall defects should be treated using a multidisciplinary approach, and NPWT can be used. This therapy can also be used during pregnancy. Vaginal delivery is preferred because it reduces the risk of further formation or deepening of the abdominal wall defect after a sufficient time interval from the start of the treatment. This complex case with a literature review of surgical complications in pregnancy treated with NPWT therapy highlights the advantage of a multidisciplinary approach.

## Synopsis

Vaginal delivery should be considered during simultaneous treatment with negative pressure therapy in situ of surgical site infection with wound dehiscence.

## 1 BACKGROUND

Infectious complications during pregnancy involve various conditions, including surgical site infections (SSI). These are particularly common after cesarean deliveries but can also occur following a laparotomy during pregnancy. The most common reason for a laparotomy during pregnancy is appendicitis.^1^

Appendicitis occurs in 100 to 223 new cases per 100 000 individuals per year in the general population and it is one of the most common causes of an acute abdomen. Appendicitis in pregnancy occurs in one in 181 to 1700 pregnancies, with the highest incidence in the second trimester. It accounts for two‐thirds of nontraumatic surgical emergencies during pregnancy.^1^ Diagnosing appendicitis during pregnancy is challenging. Clinical, laboratory, and radiological findings are necessary for a definite diagnosis. Typical symptoms such as vomiting, nausea, and abdominal pain can sometimes be difficult to distinguish from common pregnancy symptoms.^2^ The most common symptom of appendicitis, pain in the lower right quadrant, occurs near McBurney's point in most pregnant women, regardless of the stage of pregnancy.^3^

Appendectomy for appendicitis is one of the most common surgeries in pregnancy and accounts for approximately 63% of non‐obstetrical surgeries in pregnancy.^4^, ^5^ Due to the expanding uterus and the increased tension of the abdominal wall, there is a higher risk of wound dehiscence following the procedure associated with SSI.

Surgical site infection (SSI) is an infection occurring in the area of the body where the surgery was performed. We lack data regarding the incidence of SSI during pregnancy. However, it has been confirmed that this postoperative complication affects 3%–15% of women with a body mass index >30 kg/m^2^ following cesarean delivery.^6^, ^7^ Preventive measures to avoid SSI, according to National Institute for Health and Care Excellence (NICE) guidelines, include preoperative showering the day before or just prior to surgery and nasal decolonization (consider nasal mupirocin in combination with a chlorhexidine body wash before procedures where *Staphylococcus aureus* is a likely cause of SSI). Hair removal, if necessary, should only be done with electric clippers with a single‐use head on the day of surgery, as the use of razors increases the risk of SSI. Both patients and staff should wear non‐sterile theater attire in all areas where operations are performed. A prophylactic dose of antibiotics should be administered to the patient before surgery. Intraoperative measures include thorough hand disinfection by the staff, disinfection of the surgical field, and the use of sterile drapes and surgical gloves. Intracavity lavage or wound irrigation is not recommended to reduce the risk of SSI.^8^ The choice of an appropriate skin incision is crucial during pregnancy, where a low transverse suprapubic incision shows a significant decrease of 59% in the odds of developing any wound complication.^6^ Data indicates that vertical incisions performed during cesarean sections are associated with more frequent pain and postoperative complications.^9^ Postoperative care should include the use of an aseptic non‐touch technique for changing or removing surgical wound dressings, using sterile saline for wound cleansing up to 48 h after surgery, and advising patients that they may safely shower 48 h after surgery. Topical antimicrobial agents should not be used on the surgical wound by primary intention. In the event of SSI development, appropriate staff response is required to manage secondary intention wound healing. This requires appropriate interactive dressings, using antibiotic therapy that covers the most likely pathogens, meticulous and proper debridement without the use of EUSOL and gauze, dextranomer, or enzymatic treatments. The use of a systematic and multidisciplinary approach, including consultation with a tissue viability nurse, is advised.^8^

One of the potential therapeutic solutions in the case of SSI development includes negative pressure wound therapy (NPWT), also called vacuum‐assisted closure (VAC therapy).

Negative pressure wound therapy refers to a wound dressing system that improves healing by applying controlled negative pressure to the wound surface. The VAC system consists of an open‐pore polyurethane ether foam sponge, a semi‐occlusive adhesive cover, a fluid collection system, and a suction pump.^10^ The sponge is trimmed by the surgeon to match the wound size and then placed into the wound. Subsequently, the sponge is covered with an adhesive dressing, which secures it in the wound. A fenestration is made in the dressing, and a tube is inserted, connecting the sponge to the drainage canister.

The pump creates negative pressure, which is evenly distributed across the wound surface by the sponge. The pressure can be adjusted within the range of 50 to 125 mm Hg.^10^

The application of NPWT has several effects on the wound.^10^, ^11^ It increases local blood flow, which is particularly noticeable with intermittent negative pressure application. By alternating 5 min on and 2 min off, the maximal increase in local blood flow can be maintained indefinitely, as well as increased granulation tissue formation, with a 103.4 ± 35.3% mean increase in granulation tissue compared with control wounds. This is higher than with continuous negative pressure application, which results in a 63 ± 26.1% increase.^11^ NPWT also influences bacterial reduction in the wound. The decrease in bacteria count is twice as fast compared to normal wound healing due to a combination of increased blood flow, the removal of factors that slow wound healing, and the extraction of excess interstitial fluid during edema formation. This process reduces the distance between cells in the wound, facilitating tissue oxygenation, nutrient transport, and transport of new immune cells.^10^

The application of NPWT therapy offers several advantages compared to standard wound care. While conventional care might require multiple dressings per day, NPWT therapy requires dressing changes only every 2–5 days, depending on the location. Additionally, the NPWT therapy can be easily customized by adjusting the sponge size. A significant advantage is the markedly increased healing rate. The high cost compared to standard wound care is a drawback; however, one could argue, that some new therapeutic materials are also expensive.^12^ From the patient's perspective, a major benefit is the closed system, which prevents wound exudate and potential contamination of clothing. The wound is also covered, reducing the risk of further infections, allowing the patient to maintain a certain level of mobility.

Vacuum‐assisted closure therapy has numerous indications. It is applicable for burns, abdominal wounds, extracutaneous fistulae, open sternal wounds, skin graft fixation, dermal substitutes, and lower limb trauma.^13^ A prerequisite for NPWT application is thorough wound debridement before therapy to ensure adequate blood supply to the wound bed and to avoid the risk of promoting a deeper or systemic infection.^10^ Absolute contraindications for VAC therapy are presence of malignant tissue, exposed vital structures, such as organs, blood vessels, or vascular grafts, as NPWT increases the risk of tissue erosion, which can result in enteric fistula or hemorrhage. Relative contraindications include ischemic wounds, where NPWT offers no benefit, ongoing infection, or devitalized tissue, where careful wound debridement is necessary before therapy, fragile skin, and adhesive allergy.^14^

Negative pressure wound therapy is a highly effective method in the treatment of dehiscent, infected, and non‐healing wounds where the wound cannot be primarily closed due to infection, skin tension, or swelling.^15^ Since its introduction in 1994 in Europe and 1995 in the USA, the method has become widely used in a number of surgical disciplines with very good results.^16^ The use of NPWT in gynecology and especially during pregnancy is rare.

In this case study, we applied the V.A.C. ATS® Therapy System to a pregnant patient with an early dehiscent wound after appendectomy in the 30th week of pregnancy.

## 2 CASE PRESENTATION

A 34‐year‐old patient in the 29th week of pregnancy was transferred to our facility for lower abdominal pain lasting 2 days with elevated inflammatory markers without any gastrointestinal symptoms. Acute appendicitis was strongly suspected after the examination, with the possibility of ongoing perforation. The patient had an uncomplicated vaginal birth 2 years previously, did not suffer from any diseases, and had not undergone any abdominal surgery in the past. Current pregnancy was without any complications with adequate fetal growth. Due to the advanced stage of pregnancy, the size of the uterus and the suspected gangrenous peritonitis, a laparotomic approach to the abdominal cavity from McBurney's was decided upon. Prior to the surgery, the patient was given intravenous prophylactic antibiotics 3 g of ampicillin/sulbactam intravenously. Perforation of the appendix with a small amount of pus in the abdominal cavity was diagnosed perioperatively. The surgery was without any complications, the fascia was sutured with PDS 1–0, and skin was sutured with non‐absorbable 2–0 Nurolon run with single interrupted sutures, and a Penrose drain was placed next to the caecum.

Immediately after the surgery, the viability of the fetus was verified, and Magnesium sulphate was administered intravenously for incipient uterine contractions. Potassium intravenously was also given to substitute the hypokalemia. Intravenous antibiotic therapy of ampicilin/sulbactam 3 g was continued every 6 h due to high inflammatory parameters postoperatively (white blood cell count was 16 × 10^9^/L and C‐reactive protein showed 225 mg/L). The patient was put on a dysphagic diet from the second day after the surgery, which the patient initially tolerated well.

On the third postoperative day, the patient developed paralytic ileus. Due to the deteriorating condition of the patient, a nasogastric tube and a central venous catheter were inserted with subsequent administration of parenteral nutrition and intensive substitution of potassium.

On the fifth postoperative day, there was a renewed increase in inflammatory parameters. *Escherichia colli* sensitive to all antibiotics and *Pseudomonas aeruginosa* sensitive only to meropenem and amikacin were cultured. Therefore, the empiric therapy was changed to a combination of Piperacillin/tazobactam 4.5 g and metronidazole 500 mg intravenously with a good effect. A greenish fluid was flowing from the still present paracecal abdominal passive drain. The attending general surgeon performed a debridement of the wound with Betadine, dissolved two‐thirds of the length of the suture, and removed the drain.

**FIGURE 1:**
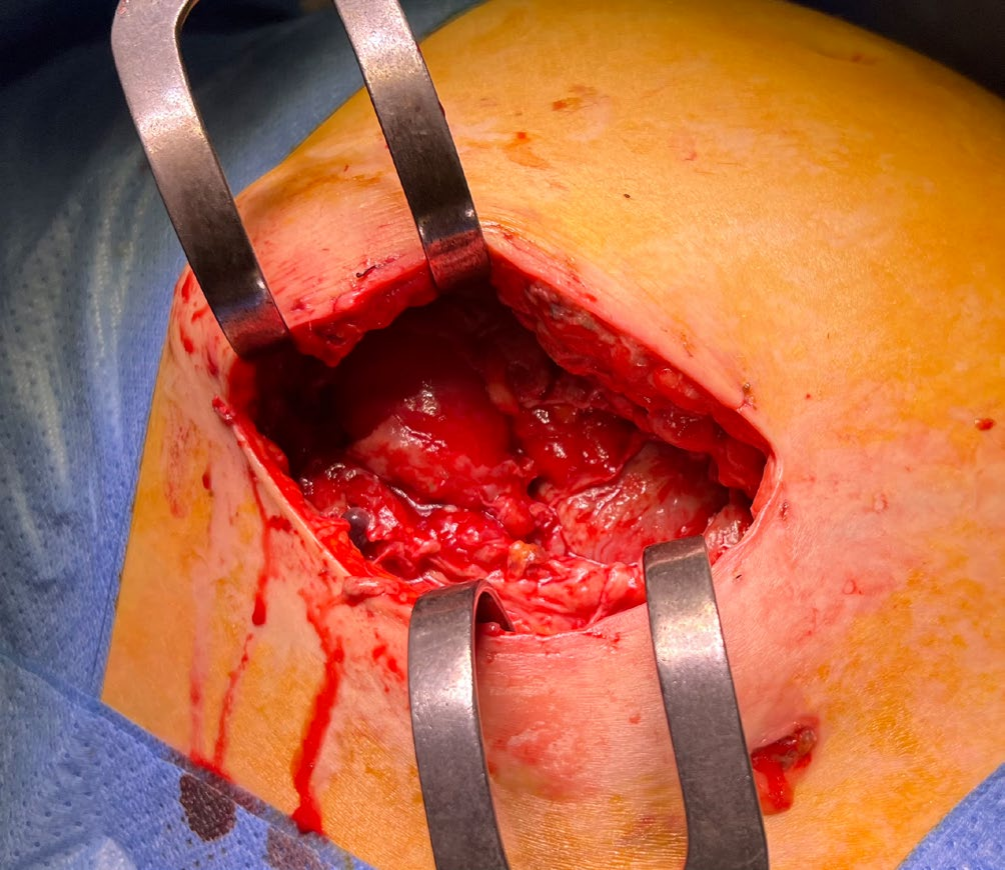
Wound dehiscence from McBurney's incision for acute appendicitis. At the base, the rest of the fascia affected by purulent fasciitis; the uterus and the cecum can be seen.

**FIGURE 2:**
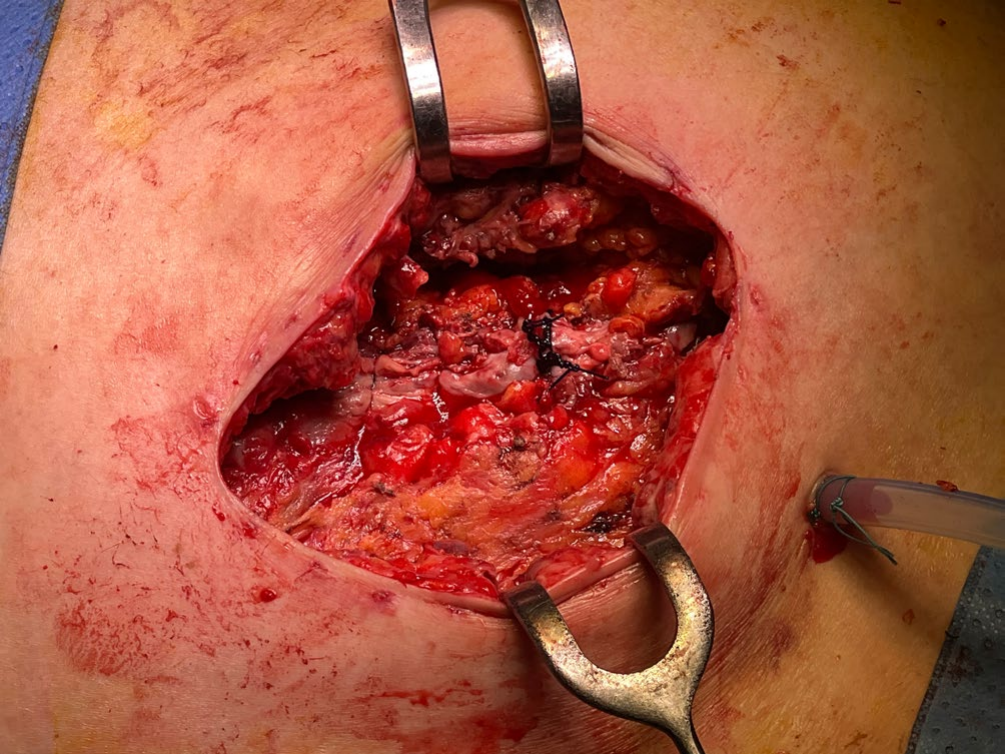
Treated wound after dehiscence. Necrotic tissues were removed, and the fascia was sutured using PDS LOOP 1–0. In the lower pole, a drain is inserted into the cecal region.

**FIGURE 3:**
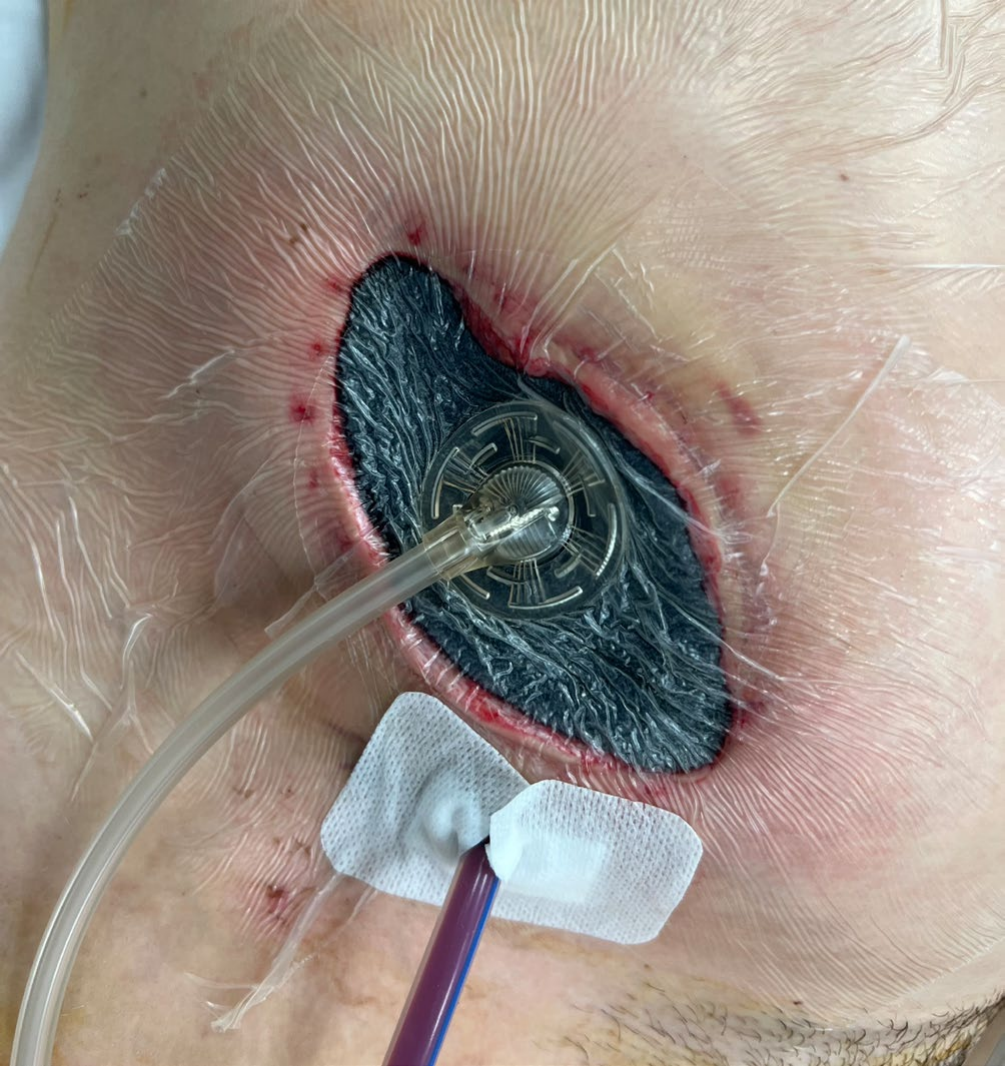
Loaded (V.A.C. ATS® Therapy System) immediately after wound treatment with a negative pressure of 50 mmHg.

On the eighth postoperative day, there was a complete dehiscence of the surgical wound in the entire length of 9 × 3 cm with purulent fasciitis and degradation of the fascia. The wound bed was made up of cecum and the pregnant uterus (Figure 1). After wound debridement, only the fascia was sutured with a continuous suture with PDS LOOP 1–0 (Figure 2) and a VAC system with a negative pressure of 50 mmHg was loaded on the sutured fascia (Figure 3).

Further hospitalization was without complications. The patient was provided with continued antibiotic therapy, low molecular weight heparin, and magnesium therapy throughout her hospitalization. The V.A.C. system was changed every 3 days with local anesthesia. Six days after fascia resuture, the patient was discharged to home care.

The patient underwent an examination every 4–6 days to replace the sponge of the V.A.C. system. The fetus was monitored by ultrasound every 7–10 days until the delivery in the outpatient clinic for high‐risk pregnancies.

At 38 weeks and 3 days of gestation, the patient was admitted for vaginal bleeding and regular contractions with a cervical score of 7. After an ultrasound examination, risk assessment was made, and due to the rapid progression of the Bishop score, it was decided to deliver vaginally. Three hours after the admission, the patient had a spontaneous delivery of a baby boy of 2860 g, with an Apgar score of 8‐9‐9 without a perineal injury. The patient did not report any pain at the site of V.A.C. therapy during delivery.

**FIGURE 4:**
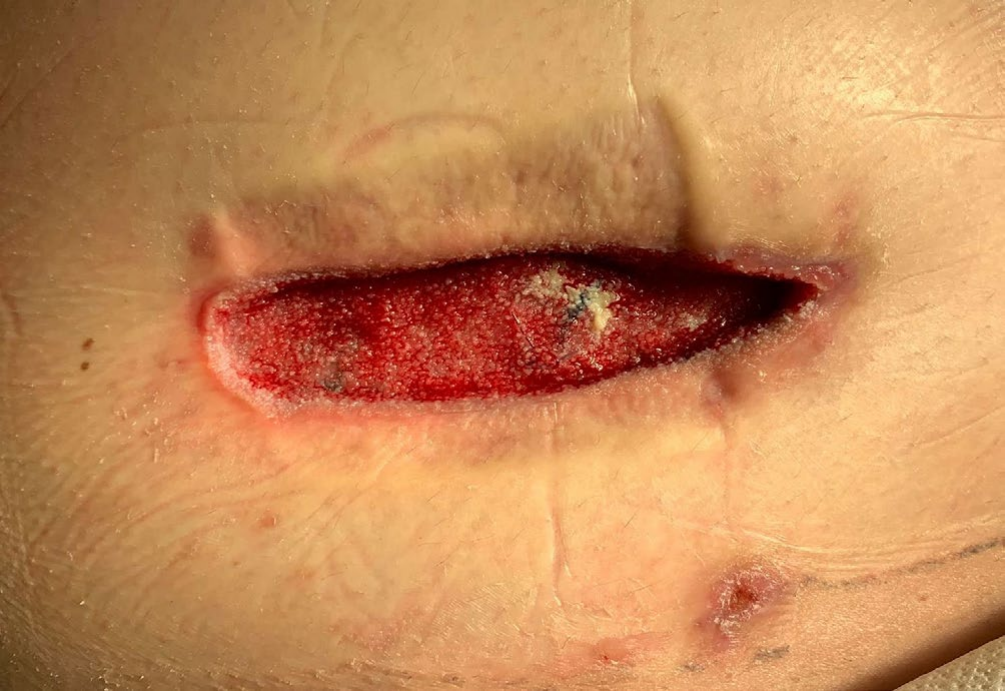
The photo was taken between the negative pressure wound therapy (NPWT) dressings (V.A.C. ATS® Therapy System).

On the third day after the delivery, the removal of NPWT therapy was performed by a surgeon under analgosedation with definitive suture of the wound. The fascia was completely healed, and the wound bed was made up of granulation tissue (Figure 4). Subcutaneous tissue suture was performed using individual sutures, an EasyFlow drain was inserted into the base, and the skin was sutured with Algower's sutures. A day later, the drain was removed, and the patient was discharged to home care.

Three months after the delivery, the patient's wound was healed by a secondary scar. The patient did not report any gynecological problems in the form of dyspareunia or vaginism. Numbness and occasional pulling in the scar area persisted.

Prior to this case report, we obtained a signed consent form from a patient, and this form is being held with our records.

## 3 DISCUSSION

This case report presents a very complex case of a complication after acute appendicitis in pregnancy with a unique approach to solving the problem due to the advanced week of pregnancy without the need for cesarean section. Correct diagnosis and safe treatment of acute appendicitis in pregnancy can be very difficult due to the enlarged uterus.^17^

The standard treatment approach for acute appendicitis is an emergency surgery.^18^ Alternatively, non‐operative treatment with administration of antibiotics entails a high risk of failure with subsequent, mostly open surgical revision. Pregnancy is not a reason to delay a correctly indicated surgery.^19^ The surgical approach can be laparoscopic or open, where laparoscopy can be more beneficial for the patient due to the shortened hospitalization and a smaller surgical wound.^20^ It is always necessary to consider the week of pregnancy and the experience of the surgeon because there is a higher risk of perioperative damage to the uterus, especially in the third trimester. The Society of American Gastrointestinal and Endoscopic Surgeons and the European Association for Endoscopic Surgery suggest that a laparoscopic appendectomy should be used for acute appendicitis when the fundus of the uterus is below the umbilicus.^17^ As in our case, when the fundus of the uterus was above the umbilicus, indications for a laparoscopic appendectomy fully depended on the surgeon's experience and preference.^17^

In our case, an open appendectomy was chosen due to the clinical condition of the patient with high inflammatory parameters. Eight days after the surgery, a complete dehiscence of the wound appeared due to an infection probably caused by gangrenous appendicitis and induced peritonitis after perforation of the appendix.

We decided to implement NPWT therapy due to the increasing size of the uterus causing skin tension and to simplify wound care. Before each sponge change, meticulous wound debridement was performed. However, early suturing was not undertaken due to the risk of recurrent wound dehiscence. A clear advantage for the patient was the dressing changes every 4–6 days, which allowed her to care for her 2‐year‐old child with minimal restrictions. NPWT therapy was set to the lowest possible pressure without any adverse effect to the fetus during periodical OB/GYN examinations. The replacement of a VAC system is often performed under general anesthesia in non‐pregnant women due to pain. In our case, for maximum protection of the fetus, the replacement of the VAC system was performed under local anesthesia and in one case under analgosedation.

Another aspect considered was the delivery method of the child, as we were concerned about possible numerous adhesions and abscesses in the abdominal cavity and the risk of intraoperative complications in the event of a cesarean section following the previous paralytic ileus.

Cases of NPWT use in pregnancy are extremely rare, and only four cases have been described worldwide.^21^, ^22^, ^23^, ^24^ To our knowledge, none of the cases describe the beginning of the therapy from the 30th week of pregnancy until the delivery. In two cases, the baby was delivered vaginally with subsequent definitive treatment of the wound after birth,^21^, ^24^ and disintegration of the fascia was described only in one of the cases.^22^ At present, the use of the V.A.C. system in pregnant patients is very rare, and therefore there is not enough information about possible adverse effects. In the described cases, negative effects on the fetus have not been observed.^21^ This is also confirmed by our experience, when the fetus did not show any signs of distress during examinations during pregnancy and after birth, such as fetal growth restriction, congenital anomalies, or poor neonatal adaptation.

Negative pressure wound therapy also has a role in obstetrics postpartum. Most documented cases of VAC system use focus on its application in patients after delivery, addressing issues such as obese patients following cesarean sections.^25^, ^26^ Case reports also mention the use of NPWT therapy in cases of abdominal compartment syndrome caused by amniotic fluid embolism, where the system was used for fluid drainage and abdominal decompression.^27^ Additionally, there has been a reported case of wound dehiscence after an episiotomy that successfully healed following the application of the NPWT system^28^, ^29^ and a case report of necrotizing fasciitis of the breast in a pregnant woman.^23^

A randomized clinical trial conducted on 1624 women demonstrated that prophylactic use of NPWT does not reduce the risk of SSI, which does not support its standardized application before the development of postoperative complications.^30^ This claim is challenged by a meta‐analysis of nine studies evaluating SSIs in 5522 patients. According to the statistical calculations, the rate of SSIs with the use of prophylactic VAC therapy in obese patients was significantly lower than with standard dressings (RR = 0.71; 95% CI = 0.56, 0.89). However, the authors note that the overall effect remains uncertain due to the inconsistency in the Bayesian meta‐analysis (RR of 0.95; 95% CI, 0.81 to 1.13). Another meta‐analysis found no differences in wound morbidity (cohort *n* = 2200; risk ratio, 1.19; 95% CI, 0.88–1.63; *I* ^2^ = 66.1%) or SSIs (randomized controlled trial *n* = 1262; risk ratio, 0.90; 95% CI, 0.63–1.29; *I* ^2^ = 0).^26^ Whitty analyzed the cost‐effectiveness of NPWT therapy compared to standard measures and reported only a 20% chance of it being more economically advantageous.^12^ The findings described the above suggested that VAC therapy should not be routinely implemented to prevent SSI in this population.

The application of the VAC system in the treatment of episiotomies was prompted by slow healing with conventional wet dressings.^28^, ^29^ In both cases, the treatment lasted up to 2 weeks with selectively administered antibiotic therapy. NPWT was applied to acute wounds in both instances, with one case involving the development of a rectovaginal hematoma and the other an SSI. The therapy resulted in successful defect healing with good cosmetic outcome and without the development of additional complications, such as dyspareunia or urinary or fecal incontinence.^28^, ^29^ This therapy offers advantages in facilitating healing in areas prone to moisture and maceration. Another benefit is the ability to care for the newborn, as was the case in our situation. Unlike our case report, where the therapy was chosen primarily to treat SSI, this treatment was selected to accelerate wound healing. We opted for this therapy mainly due to the necessity of treating SSI and the increasing tension of the abdominal wall and skin caused by the growing uterus.

Finally, one case reports the use of NPWT therapy during pregnancy for tnecrotizing fasciitis at 33 weeks of gestation. Due to the size of the defect, VAC therapy was chosen and continued after delivery for a total duration of 2 months,^23^ even though there was no disruption of the abdominal wall. Similar to our case, NPWT therapy enabled the pregnancy to reach full term. This supports our experience, along with findings from other authors,^21^, ^22^, ^24^ that NPWT therapy can prevent preterm delivery due to infection, SSI, or other postoperative complications.

Proper risk assessment in a multidisciplinary team is needed to decide on the method of management of labor. In our case, we verified the thickness of the abdominal wall by ultrasound and evaluated the strength and integrity of the wound during dressing changes, and in cooperation with general surgeons, we concluded that delivery can be performed vaginally with a low risk of wound dehiscence. This evaluation assumed a long treatment time after the defect had occurred, which allowed the formation of a solid wound bed and sufficient healing of the dehiscence. A sufficient time interval from the start of NPWT therapy is necessary for wound healing if considering the possibility of vaginal delivery. We consider it to be at least a full 3 weeks of therapy. In case of insufficient time delay from the start of NWPT therapy, we would perform a cesarean section due to the risk of rupture of the abdominal wall in the active second stage of labor.

## 4 CONCLUSION

Appendectomy for acute appendicitis is the most common non‐gynecological surgery in pregnancy. There is a risk of wound dehiscence with subsequent SSI due to infectious surgery and an expanding uterus. Our unique experience shows that in these extreme cases, long‐term use of NPWT therapy in pregnancy could be very beneficial for the woman and safe for the fetus. NPWT therapy used during pregnancy to treat site infections can prevent premature delivery and, if applied on time, it also enables to vaginal delivery.

## AUTHOR CONTRIBUTIONS

Jan Zapletal – Conceptualization, Methodology, Investigation, Figures, Writing – original draft. Borek Sehnal Ph.D. – Conceptualization, Investigation, Writing – review and editing. Radim Dvorak – Conceptualization, Investigation, Figures, Writing – review and editing. Miroslav Drienko – Writing – editing. Radovan Vlk – Writing – review and editing. J. Michael Halaska‐ Data curation, Writing – review and editing. Lukas Rob – Conceptualization, review and editing.

## FUNDING INFORMATION

This study was supported by the Cooperation program, Maternal and Childhood Care 207 035, Third Faculty of Medicine, Charles University in Prague.

## CONFLICT OF INTEREST STATEMENT

The authors declare that they have no conflict of interest.

## Data Availability

Data sharing is not applicable to this article as no new data were created or analyzed in this study.
